# Healthcare Staff Wellbeing, Burnout, and Patient Safety: A Systematic Review

**DOI:** 10.1371/journal.pone.0159015

**Published:** 2016-07-08

**Authors:** Louise H. Hall, Judith Johnson, Ian Watt, Anastasia Tsipa, Daryl B. O’Connor

**Affiliations:** 1 School of Psychology, University of Leeds, Leeds, West Yorkshire, England; 2 Yorkshire Quality and Safety Research Group, Bradford Institute for Health Research, Bradford, West Yorkshire, England; 3 Department of Health Sciences, University of York, York, North Yorkshire, England; 4 Leeds City Council, Leeds, West Yorkshire, England; University of Stirling, UNITED KINGDOM

## Abstract

**Objective:**

To determine whether there is an association between healthcare professionals’ wellbeing and burnout, with patient safety.

**Design:**

Systematic research review.

**Data Sources:**

PsychInfo (1806 to July 2015), Medline (1946 to July 2015), Embase (1947 to July 2015) and Scopus (1823 to July 2015) were searched, along with reference lists of eligible articles.

**Eligibility Criteria for Selecting Studies:**

Quantitative, empirical studies that included i) either a measure of wellbeing or burnout, and ii) patient safety, in healthcare staff populations.

**Results:**

Forty-six studies were identified. Sixteen out of the 27 studies that measured wellbeing found a significant correlation between poor wellbeing and worse patient safety, with six additional studies finding an association with some but not all scales used, and one study finding a significant association but in the opposite direction to the majority of studies. Twenty-one out of the 30 studies that measured burnout found a significant association between burnout and patient safety, whilst a further four studies found an association between one or more (but not all) subscales of the burnout measures employed, and patient safety.

**Conclusions:**

Poor wellbeing and moderate to high levels of burnout are associated, in the majority of studies reviewed, with poor patient safety outcomes such as medical errors, however the lack of prospective studies reduces the ability to determine causality. Further prospective studies, research in primary care, conducted within the UK, and a clearer definition of healthcare staff wellbeing are needed.

**Implications:**

This review illustrates the need for healthcare organisations to consider improving employees’ mental health as well as creating safer work environments when planning interventions to improve patient safety.

**Systematic Review Registration:**

PROSPERO registration number: CRD42015023340.

## Introduction

Research suggests that 16.6% of all hospital inpatient episodes in Australia and 3.7% in America lead to harmful adverse events, and in primary care, 1 in 20 prescriptions contain an error[1, 2]. In total, errors are estimated to cost the NHS £1.3 billion in litigation costs, and £2 billion in additional bed days annually[1]. Alarmingly, these statistics are likely to be an underestimate due to the complexity of trying to capture errors and adverse events within such settings.

Many factors, latent and active, system and individual, interact to cause patient safety incidents. Human factors are important contributors, and recent research indicates an important role for staff wellbeing[3–5]. Wellbeing can be conceptualised as a spectrum, with flourishing, happiness and high wellbeing at one end, and elevated depression, anxiety and low wellbeing at the other[6]. Example measures for wellbeing include the Hospital Depression and Anxiety Scale[7], the General Health Questionnaire[8], stress measures such as the Perceived Stress Scale[9], and the Positive and Negative Affect Schedule[10]. Burnout, a conceptually different variable from wellbeing, also has implications for patient safety. The burnout concept was originally developed amongst healthcare staff and is a ‘state of vital exhaustion’[11] in response to chronic organisational stress, which results in feelings of work-related exhaustion (Emotional Exhaustion; EE), depersonalisation (DP), and reduced personal accomplishment (PA)[12]. Although both wellbeing and burnout may be linked with patient safety, the current literature suffers from three limitations. First, the results of studies investigating the association between wellbeing or burnout and patient safety have been equivocal. Whilst several studies have reported an association, this finding has not always been replicated[3, 13–17]. Second, burnout has often been treated as a proxy measure for wellbeing, but the determinants, symptoms and consequences of burnout and wellbeing are distinct, and it is unclear which is more reliably associated with patient safety [18]. Third, the mechanisms underlying the association between these variables and patient safety are unclear.

Research on these associations is imperative now more than ever, due to pressures upon healthcare service budgets causing growing concerns around working conditions and the wellbeing of healthcare staff. In the UK, the financial pressures on the NHS are impacting staffing levels, causing unmanageable workloads and subsequently impacting doctors’ morale and stress levels[19–21]. Similar pressures are evident in health systems elsewhere, for example, in the US, there is growing concern over a caregiver shortage occurring, due to population increases, chronic disease growth, and increased life expectancies[22]. In light of this, we conducted a systematic review to investigate the extent to which wellbeing and burnout are associated with patient safety.

## Aims and Objectives

The overarching aim of the review was to synthesize existing research investigating the association between wellbeing and/or burnout in healthcare professionals with the safety of patient care. The review had three specific aims:

1. To explore the association between wellbeing in health care professionals and patient safety.
2. To explore the association between burnout in health care professionals and patient safety.
3. To explore the studies that measure both wellbeing and burnout in relation to patient safety.

## Method

The protocol for this systematic review was registered in advance on PROSPERO, in which the inclusion criteria and methods of analysis were specified: registration number: CRD42015023340. The protocol can be accessed here: http://www.crd.york.ac.uk/PROSPERO/display_record.asp?ID=CRD42015023340. This review has not been subject to meta-analysis due to the heterogeneity of the measures. This review was conducted in line with the Preferred Reporting Items for Systematic Reviews and Meta-Analyses (PRISMA) guidelines[23], see S1 File.

### Search strategy

Four electronic bibliographic databases were last searched on the 20/07/2015, see Table 1, along with reference searching of all eligible articles. Authors of inaccessible articles were contacted to attain full texts. Both MeSH terms and keyword terms were used in a multi-field search, based on terms commonly used within systematic reviews in the fields of patient safety, and wellbeing and burnout.

**Table 1:** Electronic databases searched and number of results.

Database	Papers Identified
PsycINFO (1806 –July 2015)	124
MEDLINE (1946 –July 2015)	4480
Embase (1947 –July 2015)	7139
Scopus (1823 –July 2015)	288
Total	12031

Papers were searched for those containing at least one term from each of the following blocks (although MeSH terms varied slightly between databases): (health personnel) AND (well?being OR occupation* stress* OR burnout OR mental health OR “quality of life”) AND (medical error OR patient safety OR quality of care OR error?). For an example of full search terms used for one of the databases, see S2 File.

### Study selection

**Fig 1:**
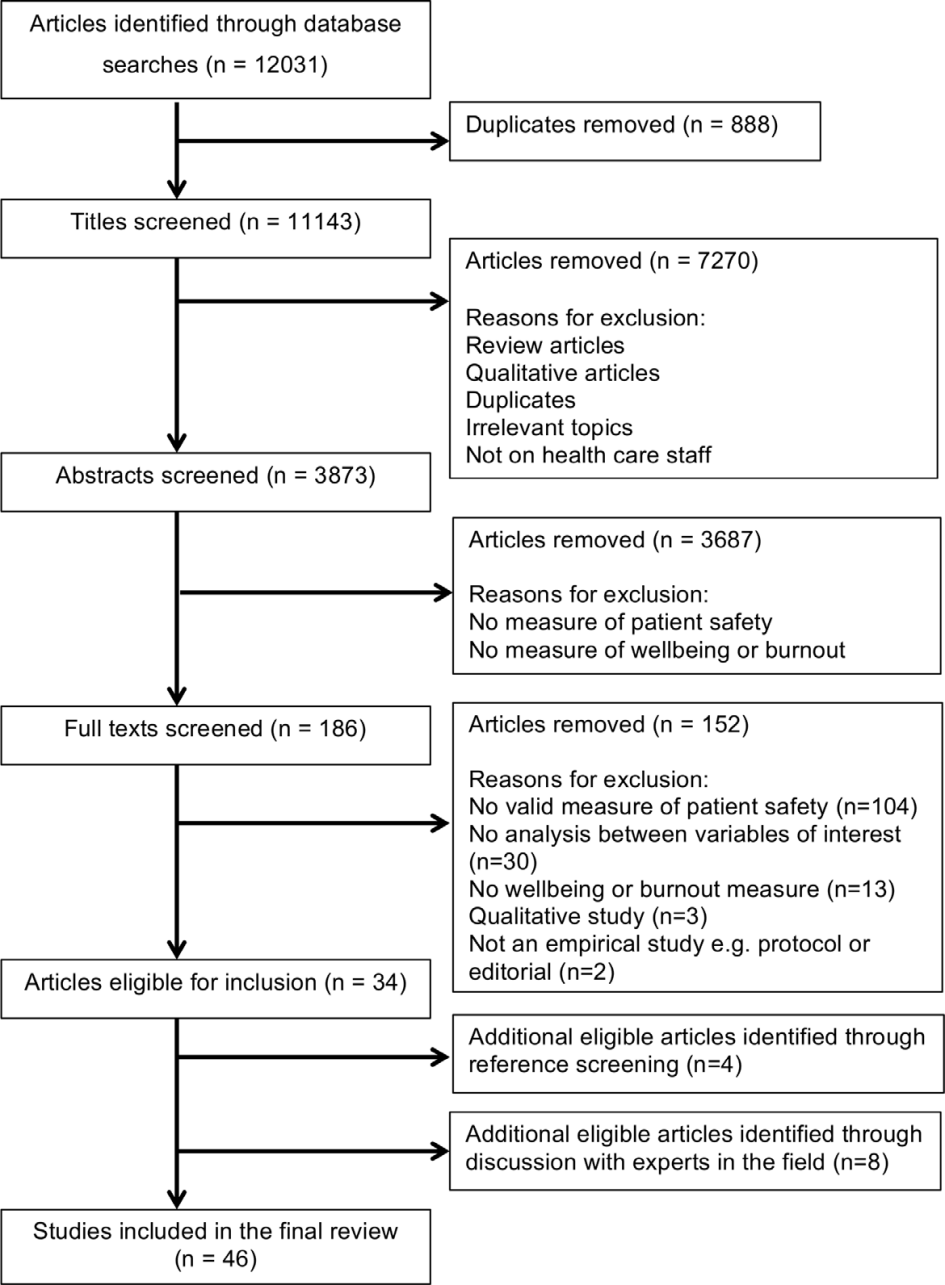
Flow chart documenting the screening process.

A flow chart documenting the selection process can be viewed in Fig 1. After duplicates were removed, titles were screened by one author (LH). A second author (AT) checked 10% of the excluded titles, with 100% agreement. All abstracts were screened by LH, and double screened by the remaining authors (JJ, DOC, AT, IT) to check agreement at this stage. Non-agreement was resolved through discussion between at least two authors. Full texts were screened by LH, who ensured inclusion of any questionable articles for further consideration and discussion with a second author at the data extraction stage. Any differences in opinion between the authors regarding article eligibility, or key criteria during data extraction, were resolved through discussion with a third member of the research team when no consensus could be achieved. Articles were screened for eligibility against the following criteria.

#### Inclusion criteria

Peer reviewed observational, cross-sectional and prospective studies that were published and included both a measure of wellbeing and/or burnout in healthcare staff, and a measure of patient safety were included. No restrictions on the year of publication were imposed, but only articles written or translated into English were eligible. Qualitative research along with case studies, review articles, editorials, letters, conference abstracts, books, theses and opinions were excluded. Studies that only included healthcare staff that do not directly deal with patients (e.g. hospital receptionists) were excluded.

During the abstract screening process, additional exclusion criteria were applied by LH and JJ, due to a large number of studies measuring variables that were related to patient safety, but did not satisfy the author’s definitions of the variables of interest. The following terms, if not measured alongside a valid measure of safety resulted in rejection of the article: Job or work satisfaction, workability, motivation, productivity, and attitudes towards work. Additionally, litigation or legal action, without any explicit mention of the variables of interest was not included.

### Data extraction and quality assessment

The first 10% of eligible articles at this stage went through a standardized data extraction and quality assessment process (S3 and S4 Files) by two authors (LH and AT) to reduce bias and ensure reliability. The remaining 90% of articles were extracted solely by LH. The data extraction form was refined during the extraction of the first few articles to ensure the forms were comprehensive.

A quality assessment tool was developed for this review, based on the criteria that were transferable to non-randomized clinical trial studies from the COCHRANE risk of bias tool, along with additional categories defined *a priori* by the authors to assess reliability and validity of the measures used (S4 File). Each article was assessed using the quality assessment tool and then all articles were summarized together to give an impression of the overall quality of the studies included in the review.

## Results

Forty-six studies were deemed eligible for inclusion in this review, and they were subsequently grouped based on whether they measured wellbeing or burnout, or both.

### Descriptive statistics and study characteristics

Nineteen studies measured burnout, sixteen measured wellbeing, and the remaining eleven included both a measure of burnout and of wellbeing. Of the burnout studies, the vast majority of studies (*n* = 24) used some variant of the Maslach Burnout Inventory (MBI) [24], such as the MBI-Human Services Survey, the EE scale of the MBI, or an international variation of the MBI. Alternative measures were; Shirom-Melamed’s Burnout Scale, Copenhagen Burnout Inventory (CBI), Physician Well-Being Index, a single question approach and a symptom-based stress survey. The wellbeing measures were far more varied and included; General Health Questionnaire (GHQ), Harvard National Depression Screening Day Scale (HANDS), linear stress scales, Quality of Life scales, and emotional distress, among others. For a full list of measures and other study characteristics, see S1 Table. Most of the measures used were pre-existing, validated and reliable measures.

The most common approach to measuring patient safety was to use a question on the frequency of self-perceived errors over a particular time frame, ranging from the previous four weeks to the past year. Other subjective and self reported measures included stating the health professional’s accident propensity (from ‘not at all’ to ‘very likely’) and their perceived likelihood of making errors. One study used a hand held computer device to gather medication events in real time[25]. Six studies included objective measures of error, using chart audits, checking official error reports, and conducting observations of staff[5, 16, 26–29]. Fahrenkopf and colleagues[28], and Garrouste-Orgeas and colleagues[29] were the only studies to use both objective and subjective measures of error. Although Welp and colleagues[30] measured mortality rates and length of stay alongside a subjective measure of patient safety, these objective measures were not within our criteria for patient safety outcomes.

The studies were conducted across 16 different countries and six continents, with a large proportion being based in America (*n* = 19). Most (*n* = 33) utilised a cross-sectional survey design, with only nine using a prospective cohort study methodology. The most commonly studied profession was nurses (*n* = 24 studies), followed by physicians (*n* = 7). The remaining study samples consisted of pharmacists (*n* = 2), a variety of hospital staff (*n* = 2), paramedics (*n* = 1), surgeons (*n* = 2), anaesthetists (*n* = 1) and doctors still in some form of training (*n* = 8). Only one study included primary care physicians, and they were grouped in with the hospital based staff in all the analyses.

### Wellbeing findings

Of the articles measuring wellbeing, over half (16/27, 59.3%) found that poor wellbeing, as measured using a variety of definitions (depression, anxiety, job stress, mental health, distress), was associated with poorer patient safety [3–5, 17, 25, 29, 31–40]. An additional six studies (22.2%) found some sort of relationship between wellbeing and patient safety, but with only some subscales of the wellbeing measures or safety measures correlating[27, 28, 41–44]. Tanaka and colleagues’ prospective cohort study in Japan[4] found that higher depression scores were significantly associated with more near misses, but not with adverse events, as measured through frequency of self-perceived error in the previous 6 months. Of note is that both Houston and Allt’s study[41], and Niven and Ciborowska’s study[42] found that anxiety, but not depression, was significantly associated with errors, despite using different measures of anxiety, depression and errors from each other.

Five studies found no correlation between wellbeing and patient safety [13, 16, 45–47]. However, one of these, Dorrian and colleagues’ 2006 study[13], was only a pilot study, with a sample size of 23. Their full study in 2008, however, did find that stress significantly predicted error, suggesting that the pilot study was underpowered.

Amongst the studies of wellbeing, Hammer and colleagues’ cross-sectional study of 374 paramedics in the United States (US)[40] was distinctive in that it indicated an association between low stress and increased error. They reported that those with less somatic distress and lower total stress scores (on the MPSS-R) made significantly more errors. This could perhaps be due to the measures used, which differ from all the other studies. The stress measure was of organisational stress, and did not measure the participants’ own stress levels. The distress measure is also questionable, as although it taps into some concepts associated with poor wellbeing, such as, “I wake up feeling tired”, others questions ask about behaviours that are not necessarily indicative of poor mental health, e.g. “I drink on the weekend to relax”.

### Burnout Findings

Similarly to the wellbeing studies, the majority of studies (21/30, 70%) measuring burnout found that more errors were significantly associated with health practitioner burnout[3, 14, 26, 30, 31, 33, 35, 37, 38, 46, 48–58]. Four studies additionally found partial associations between burnout and error[28, 59–61]. For example, Halbesleben and colleagues’[59] cross-sectional survey of nurses in the US found that higher burnout was significantly associated with a lower patient safety grade and near miss reporting frequency, but not with event report frequency. Klein and colleagues [60]found that burnout, as measured by the Copenhagen Burnout Inventory, was only significantly associated with therapeutic (OR = 2.54) and diagnostic errors (OR = 1.94) in male, but not in female surgeons in Germany.

Five studies did not find any significant associations between burnout and error [16, 27, 62–64]. Of these studies, only one of them used a full set of MBI questions, although this was a translated version[29]. The remaining studies used a single-item measure, only the EE subscale of the MBI, and a symptom-based stress survey, which although it had been previously used to measure burnout, the authors describe it as a stress, and not a burnout survey in this context [27].

### Studies measuring both burnout and wellbeing

Eleven studies measured both burnout and wellbeing in relation to patient safety outcomes[3, 16, 27–29, 31, 33, 35, 37, 38, 46]. It is these studies that may facilitate a more intricate understanding of which variable is linked with the greater risk of error. Although the majority (7/11) found that both poor wellbeing and risk of burnout were significantly associated with errors, all these studies, bar one[28], used only self-perceived errors as the outcome indicator. All but one[46] of the remaining studies that found either no link at all or only an association between either wellbeing or burnout with safety, were those that used objective measures of error, suggesting that perhaps objective measures are not sensitive enough[16, 27, 29]. Linzer and colleagues[16] conducted chart audits and found no associations between errors and wellbeing or burnout. Dugan and colleagues[27] checked hospital records for errors and found that stress scores (stress continuum scale) but not a symptom-based stress survey (a possible burnout measure) correlated with patient incidents. Finally, Garrouste-Orgeas and colleagues[29] found that in their prospective cohort study, depression was an independent risk factor for error (as assessed by chart audit), but that burnout was not. The one study that used both objective and subjective measures of error found that different measurement methods resulted in different findings for burnout than for wellbeing[28]. In this study, depressed resident doctors made significantly more errors than those who were not depressed, but only when using the objective, and not when using the subjective, measure of error. Additionally, they found that burnt-out residents made more errors than non-burnt-out residents when using subjective self-reported, but not objective, measures of error.

Four studies [29, 31, 33, 37] conducted analyses that could determine whether burnout and poor wellbeing were each independent predictors of error, or whether one explained the variance in the other. Garrouste-Orgeas and colleagues[29] concluded that burnout was not directly associated with error, even when depression was controlled for in the analysis. Depression however, was found to be an independent predictor of error. The three remaining studies [31, 33, 37] reported that both burnout and wellbeing were independent predictors of error when multivariate analyses were conducted.

### Study quality and risk of bias

**Fig 2:**
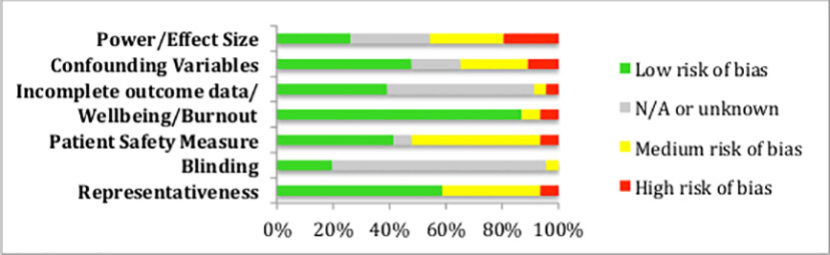
Risk of bias graph displaying the overall study quality for all 46 studies.

See Fig 2 for an overview of all the studies combined risks of bias, based on the format suggested by the COCHRANE guidelines. For separate quality rating graphs for the wellbeing and burnout studies, see S5 File.

A common concern amongst the studies with regards to quality was the measure of patient safety used, as the majority of studies used solely self-reported measures of error, which has a number of limitations including social desirability and fear of blame and retribution. This shall be discussed further in the following section.

There was not much variability in study quality/risk of bias between those that measured wellbeing and those that measured burnout. The criteria in which the burnout studies generally displayed a lower risk of bias than the wellbeing studies were representativeness (73.3% v. 44.4%), and measures of burnout/wellbeing (93.3% v. 77.7%), respectively. There were a similar number of studies demonstrating a medium to large effect size for the relationship between patient safety and burnout as there were for patient safety and wellbeing (33.3% v. 25.9%).

## Discussion

The majority of studies provided evidence that both wellbeing and burnout are associated with patient safety. In particular, poor wellbeing, as characterized by depression, anxiety, poor quality of life and stress, and high levels of burnout, were found to be significantly associated with more self-reported errors, with a smaller number of studies showing an association of these factors with objective measures of error. A similar percentage of studies found significant associations between wellbeing and error (88.9% of studies) as those for burnout and error (83.3%), indicating the importance of both variables. Studies reporting that both burnout and poor wellbeing were independently associated with poorer patient safety suggest the importance of both variables on their own [31, 33, 37]. Indeed, one study[31] found that when resident doctors suffered from both high burnout and risk of depression, they reported even more errors than those who suffered solely from burnout or depression alone. These studies signify that both staff wellbeing and burnout may be important targets for patient safety interventions. However further research is needed first to properly understand the nature of the relationships between these factors. Too few prospective studies have been conducted to definitively propose a causal relationship. West and colleagues’ study[3] attempts to clarify this, with a circular relationship between burnout and error being reported. However this circular relationship was not found for wellbeing and error, with errors significantly predicting subsequent quality of life and depression ratings, but not vice versa. An explanatory model for how wellbeing and burnout are associated is also needed, to enable the implementation of effective interventions. Fahrenkopf and colleagues’ study[28] found that 96% of depressed residents were also burnt-out, but only 25% of burnt-out residents were depressed, indicating that burnout may be a possible precursor to depression. If the findings from these studies could be extrapolated into an overarching model, it could be proposed that overworked staff become burnt-out, which may eventually lead to depression. Thus burnout and depression may manifest itself through fatigue, irritability and reduced cognitive functioning[65], all of which puts pressure on team relationships causing a poorer safety climate, and on their own individual work performance, resulting in more distanced staff, poorer quality of care and ultimately a higher risk of making errors.

### Limitations of the studies

This review is limited in its ability to determine the nature of the associations between wellbeing, burnout and patient safety, due to the limitations of the studies included. The primary limitation was the measure of safety, which is a general problem within patient safety research. The measures used often relied on self-reported errors with recall as long as a year ago, making the results vulnerable to a variety of memory biases and cognitive failures. However despite these limitations, self-reported measures do provide a number of advantages over subjective measures; they are more sensitive, they can provide information across all types of errors, and they can be measured at the individual level more easily than objective measures can. There is a lack of studies measuring both objective and subjective measures of safety, with those that measured both differing in the type of errors measured by the two different methods [28].

### Limitations of this review, and further research

This review is restricted by its exclusion of non-English language papers. Additional limitations include the possibility of a publication bias due to the exclusion of grey literature, and the inability to quantitatively determine whether wellbeing or burnout is more strongly associated with patient safety due to the heterogeneity of the wellbeing and safety measures used. However, the eligible studies were from a wide range of locations and included a variety of job roles (nurses, surgeons etc.). Future research is needed to address these issues, and interventions need to be trialed at various points of the organisational, staff and patient levels to determine where the most effective intervention for staff wellbeing and patient safety will fit.

## Conclusion

The finding that burnout and poor wellbeing are both, in the majority of studies reviewed, associated with poorer patient safety has significant implications for policymakers and management teams within healthcare settings. To deliver quality patient care, the care must first and foremost be safe, and the findings from this review suggest that staff wellbeing may play an important role in patient safety. It would seem prudent that healthcare organisations provide a work environment that fosters staff wellbeing and protects against burnout, to subsequently provide a safe service to their patients.

## Supporting Information

S1 Table Summary table of results. (PDF)

S1 File PRISMA checklist. (PDF)

S2 File Search criteria for Medline (Ovid). (PDF)

S3 File Data extraction template. (PDF)

S4 File Quality assessment tool and scoring guide. (PDF)

S5 File Quality assessment graphs grouped by wellbeing and burnout studies. (PDF)

## References

[1] Health Do. An Organisation with a Memory: Report of an Expert Group on Learning from Adverse Events in the NHS Chaired by the Chief Medical Officer: The Stationery Office London; 2000.

[2] Avery T, Barber N, Ghaleb M, Franklin BD, Armstrong S, Crowe S, et al Investigating the prevalence and causes of prescribing errors in general practice London: The General Medical Council: PRACtICe Study. 2012.

[3] West CP, Huschka MM, Novotny PJ, Sloan JA, Kolars JC, Habermann TM, et al Association of perceived medical errors with resident distress and empathy: A prospective longitudinal study. Journal of the American Medical Association. 2006;296(9):1071–8. . 16954486 10.1001/jama.296.9.1071

[4] Tanaka M, Tanaka K, Takano T, Kato N, Watanabe M, Miyaoka H. Analysis of risk of medical errors using structural-equation modelling: A 6-month prospective cohort study. BMJ Quality and Safety. 2012;21(9):784–90. 10.1136/bmjqs-2010-048330 22927491

[5] Saleh AM, Awadalla NJ, El-masri YM, Sleem WF. Impacts of nurses' circadian rhythm sleep disorders, fatigue, and depression on medication administration errors. Egyptian Journal of Chest Diseases and Tuberculosis. 2014;63(1):145–53. .

[6] Johnson J, Wood AM. Integrating Positive and Clinical Psychology: Viewing Human Functioning as Continua from Positive to Negative Can Benefit Clinical Assessment, Interventions and Understandings of Resilience. Cognitive Therapy and Research. 2016:1–15.28216800

[7] Zigmond AS, Snaith RP. The hospital anxiety and depression scale. Acta psychiatrica scandinavica. 1983;67(6):361–70. 6880820 10.1111/j.1600-0447.1983.tb09716.x

[8] Goldberg DP, Hillier VF. A scaled version of the General Health Questionnaire. Psychological medicine. 1979;9(01):139–45. 424481 10.1017/s0033291700021644

[9] Cohen S, Kamarck T, Mermelstein R. A global measure of perceived stress. Journal of health and social behavior. 1983:385–96. 6668417

[10] Watson D, Clark LA, Tellegen A. Development and validation of brief measures of positive and negative affect: the PANAS scales. Journal of personality and social psychology. 1988;54(6):1063 3397865 10.1037//0022-3514.54.6.1063

[11] Organization WH. International statistical classification of diseases and health related problems (The) ICD-10: World Health Organization; 2004.

[12] Maslach C, Jackson SE. The measurement of experienced burnout. Journal of occupational behavior. 1981;2(2):99–113.

[13] Dorrian J, Lamond N, Van Den Heuvel C, Pincombe J, Rogers AE, Dawson D. A pilot study of the safety implications of Australian nurses' sleep and work hours. Chronobiology international. 2006;23(6):1149–63. 17190702 10.1080/07420520601059615

[14] Holden RJ, Patel NR, Scanlon MC, Shalaby TM, Arnold JM, Karsh B-T. Effects of mental demands during dispensing on perceived medication safety and employee well-being: a study of workload in pediatric hospital pharmacies. Research in social and administrative Pharmacy. 2010;6(4):293–306. 10.1016/j.sapharm.2009.10.001 21111387 PMC3052977

[15] Holden RJ, Scanlon MC, Patel NR, Kaushal R, Escoto KH, Brown RL, et al A human factors framework and study of the effect of nursing workload on patient safety and employee quality of working life. BMJ quality & safety. 2011;20(1):15–24. 10.1136/bmjqs.2008.028381 PMC3058823 21228071

[16] Linzer M, Manwell LB, Williams ES, Bobula JA, Brown RL, Varkey AB, et al Working conditions in primary care: physician reactions and care quality. Annals of internal medicine. 2009;151(1):28–36. 19581644 10.7326/0003-4819-151-1-200907070-00006

[17] Suzuki K, Ohida T, Kaneita Y, Yokoyama E, Miyake T, Harano S, et al Mental health status, shift work, and occupational accidents among hospital nurses in Japan. Journal of occupational health. 2004;46(6):448–54. 15613767 10.1539/joh.46.448

[18] Schaufeli W, Enzmann D. The burnout companion to study and practice: A critical analysis: CRC press; 1998.

[19] Raymond J. BMA quarterly tracker survey, April 2014 British Medical Association 4 2014.

[20] Association BM. BMA quarterly tracker survey: Current views from across the medical profession. Quarter 2: 4 2015. April 2015.

[21] Gibson J, Checkland K, Coleman A, Hann M, McCall R, Spooner S, et al. Eight national GP worklife survey. 2015.

[22] http://www.csc.com/health_services/insights/48705-u_s_healthcare_workforce_shortages_caregivers.

[23] Moher D, Liberati A, Tetzlaff J, Altman DG. Preferred reporting items for systematic reviews and meta-analyses: the PRISMA statement. Annals of internal medicine. 2009;151(4):264–9. 19622511 10.7326/0003-4819-151-4-200908180-00135

[24] Maslach C, Jackson SE, Leiter MP. Maslach burnout inventory manual Mountain View, CA: CPP. Inc, and Davies-Black 1996.

[25] Dollarhide AW, Rutledge T, Weinger MB, Fisher ES, Jain S, Wolfson T, et al A Real‐Time Assessment of Factors Influencing Medication Events. Journal for Healthcare Quality. 2014;36(5):5–12. 10.1111/jhq.12012 23551380

[26] Cimiotti JP, Aiken LH, Sloane DM, Wu ES. Nurse staffing, burnout, and health care–associated infection. American journal of infection control. 2012;40(6):486–90. 10.1016/j.ajic.2012.02.029 22854376 PMC3509207

[27] Dugan J, Lauer E, Bouquot Z, Dutro BK, Smith M, Widmeyer G. Stressful nurses: the effect on patient outcomes. Journal of Nursing Care Quality. 1996;10(3):46–58. 8634470

[28] Fahrenkopf AM, Sectish TC, Barger LK, Sharek PJ, Lewin D, Chiang VW, et al Rates of medication errors among depressed and burnt out residents: prospective cohort study. Bmj. 2008;336(7642):488–91. 10.1136/bmj.39469.763218.BE 18258931 PMC2258399

[29] Garrouste-Orgeas M, Perrin M, Soufir L, Vesin A, Blot F, Maxime V, et al The Iatroref study: medical errors are associated with symptoms of depression in ICU staff but not burnout or safety culture. Intensive care medicine. 2015;41(2):273–84. 10.1007/s00134-014-3601-4 25576157

[30] Welp A, Meier LL, Manser T. Emotional exhaustion and workload predict clinician-rated and objective patient safety. Frontiers in psychology. 2015;5:1573 10.3389/fpsyg.2014.01573 25657627 PMC4302790

[31] de Oliveira GS Jr, Chang R, Fitzgerald PC, Almeida MD, Castro-Alves LS, Ahmad S, et al The prevalence of burnout and depression and their association with adherence to safety and practice standards: a survey of United States anesthesiology trainees. Anesthesia & Analgesia. 2013;117(1):182–93. 23687232 10.1213/ANE.0b013e3182917da9

[32] Dorrian J, Tolley C, Lamond N, van den Heuvel C, Pincombe J, Rogers AE, et al Sleep and errors in a group of Australian hospital nurses at work and during the commute. Applied ergonomics. 2008;39(5):605–13. 10.1016/j.apergo.2008.01.012 18395183

[33] Dyrbye LN, Satele D, Sloan J, Shanafelt TD. Utility of a brief screening tool to identify physicians in distress. Journal of general internal medicine. 2013;28(3):421–7. 10.1007/s11606-012-2252-9 23129161 PMC3579983

[34] Fogarty GJ, McKeon C. Patient safety during medication administration: the influence of organizational and individual variables on unsafe work practices and medication errors. Ergonomics. 2006;49(5–6):444–56. 16717003 10.1080/00140130600568410

[35] Hayashino Y, Utsugi-Ozaki M, Feldman MD, Fukuhara S. Hope modified the association between distress and incidence of self-perceived medical errors among practicing physicians: prospective cohort study. 2012. 10.1371/journal.pone.0035585 PMC3329473 22530055

[36] Pelliciotti JdSS, Kimura M. Medications errors and health-related quality of life of nursing professionals in intensive care units. Revista latino-americana de enfermagem. 2010;18(6):1062–9. 21340269 10.1590/s0104-11692010000600004

[37] Shanafelt TD, Balch CM, Bechamps G, Russell T, Dyrbye L, Satele D, et al Burnout and medical errors among American surgeons. Annals of surgery. 2010;251(6):995–1000. 10.1097/SLA.0b013e3181bfdab3 19934755

[38] West CP, Tan AD, Habermann TM, Sloan JA, Shanafelt TD. Association of resident fatigue and distress with perceived medical errors. Jama. 2009;302(12):1294–300. 10.1001/jama.2009.1389 19773564

[39] Arimura M, Imai M, Okawa M, Fujimura T, Yamada N. Sleep, mental health status, and medical errors among hospital nurses in Japan. Industrial health. 2010;48(6):811–7. 20616466 10.2486/indhealth.ms1093

[40] Hammer JS, Mathews JJ, Lyons JS, Johnson NJ. Occupational stress within the paramedic profession: An initial report of stress levels compared to hospital employees. Annals of emergency medicine. 1986;15(5):536–9. 3963532 10.1016/s0196-0644(86)80988-x

[41] Houston DM, Allt SK. Psychological distress and error making among junior house officers. British Journal of Health Psychology. 1997;2(2):141–51.

[42] Niven K, Ciborowska N. The hidden dangers of attending work while unwell: A survey study of presenteeism among pharmacists. International Journal of Stress Management. 2015;22(2):207.

[43] Park Y-M, Kim SY. Impacts of job stress and cognitive failure on patient safety incidents among hospital nurses. Safety and health at work. 2013;4(4):210–5. 10.1016/j.shaw.2013.10.003 24422177 PMC3889080

[44] Arakawa C, Kanoya Y, Sato C. Factors contributing to medical errors and incidents among hospital nurses-nurses' health, quality of life, and workplace predict medical errors and incidents. Industrial health. 2011;49(3):381–8. 21372434 10.2486/indhealth.ms968

[45] Baldwin P, Dodd M, Wrate R. Young doctors' health—I. How do working conditions affect attitudes, health and performance? Social science & medicine. 1997;45(1):35–40. 9203268 10.1016/s0277-9536(96)00306-1

[46] Shanafelt TD, Bradley KA, Wipf JE, Back AL. Burnout and self-reported patient care in an internal medicine residency program. Annals of internal medicine. 2002;136(5):358–67. 11874308 10.7326/0003-4819-136-5-200203050-00008

[47] Wilkins K, Shields M. Correlates of medication error in hospitals. Health reports. 2008;19(2):7 18642515

[48] Bao Y, Vedina R, Moodie S, Dolan S. The relationship between value incongruence and individual and organizational well‐being outcomes: an exploratory study among Catalan nurses. Journal of advanced nursing. 2013;69(3):631–41. 10.1111/j.1365-2648.2012.06045.x 22632178

[49] Block L, Wu AW, Feldman L, Yeh H-C, Desai SV. Residency schedule, burnout and patient care among first-year residents. Postgraduate medical journal. 2013;89(1055):495–500. 10.1136/postgradmedj-2012-131743 23852828

[50] Chen K-Y, Yang C-M, Lien C-H, Chiou H-Y, Lin M-R, Chang H-R, et al Burnout, job satisfaction, and medical malpractice among physicians. International journal of medical sciences. 2013;10(11):1471 10.7150/ijms.6743 24046520 PMC3775103

[51] Laschinger HKS, Leiter MP. The impact of nursing work environments on patient safety outcomes: The mediating role of burnout engagement. Journal of Nursing Administration. 2006;36(5):259–67. 16705307 10.1097/00005110-200605000-00019

[52] Prins J, Van Der Heijden F, Hoekstra-Weebers J, Bakker A, Van de Wiel H, Jacobs B, et al Burnout, engagement and resident physicians' self-reported errors. Psychology, health & medicine. 2009;14(6):654–66. 10.1080/13548500903311554 20183538

[53] Profit J, Sharek PJ, Amspoker AB, Kowalkowski MA, Nisbet CC, Thomas EJ, et al Burnout in the NICU setting and its relation to safety culture. BMJ quality & safety. 2014;23(10):806–13. 10.1136/bmjqs-2014-002831 PMC4167972 24742780

[54] Teng C-I, Shyu Y-IL, Chiou W-K, Fan H-C, Lam SM. Interactive effects of nurse-experienced time pressure and burnout on patient safety: a cross-sectional survey. International Journal of Nursing Studies. 2010;47(11):1442–50. 10.1016/j.ijnurstu.2010.04.005 20472237

[55] Williams ES, Manwell LB, Konrad TR, Linzer M. The relationship of organizational culture, stress, satisfaction, and burnout with physician-reported error and suboptimal patient care: results from the MEMO study. Health care management review. 2007;32(3):203–12. 17666991 10.1097/01.HMR.0000281626.28363.59

[56] Zander B, Dobler L, Busse R. The introduction of DRG funding and hospital nurses’ changing perceptions of their practice environment, quality of care and satisfaction: comparison of cross-sectional surveys over a 10-year period. International journal of nursing studies. 2013;50(2):219–29. 10.1016/j.ijnurstu.2012.07.008 22846589

[57] Ramanujam R, Abrahamson K, Anderson JG. Influence of workplace demands on nurses' perception of patient safety. Nursing & Health Sciences. 2008;10(2):144–50. 18466388 10.1111/j.1442-2018.2008.00382.x

[58] Squires M, Tourangeau A, SPENCE LASCHINGER HK, Doran D. The link between leadership and safety outcomes in hospitals. Journal of Nursing Management. 2010;18(8):914–25. 10.1111/j.1365-2834.2010.01181.x 21073565

[59] Halbesleben JR, Wakefield BJ, Wakefield DS, Cooper LB. Nurse burnout and patient safety outcomes nurse safety perception versus reporting behavior. Western Journal of Nursing Research. 2008;30(5):560–77. 10.1177/0193945907311322 18187408

[60] Klein J, Frie KG, Blum K, von dem Knesebeck O. Burnout and perceived quality of care among German clinicians in surgery. International Journal for Quality in Health Care. 2010;22(6):525–30. 10.1093/intqhc/mzq056 20935011

[61] Van Bogaert P, Timmermans O, Weeks SM, van Heusden D, Wouters K, Franck E. Nursing unit teams matter: Impact of unit-level nurse practice environment, nurse work characteristics, and burnout on nurse reported job outcomes, and quality of care, and patient adverse events—A cross-sectional survey. International journal of nursing studies. 2014;51(8):1123–34. 10.1016/j.ijnurstu.2013.12.009 24444772

[62] Holden RJ, Scanlon MC, Patel NR, Kaushal R, Escoto KH, Brown RL, et al A human factors framework and study of the effect of nursing workload on patient safety and employee quality of working life. BMJ Quality and Safety. 2011;20(1):15–24. 10.1136/bmjqs.2008.028381 PMC3058823 21228071

[63] Garrouste-Orgeas M, Perrin M, Soufir L, Vesin A, Blot F, Maxime V, et al The Iatroref study: medical errors are associated with symptoms of depression in ICU staff but not burnout or safety culture. Intensive Care Medicine. 2015;41(2):273–84. 10.1007/s00134-014-3601-4 25576157

[64] Kirwan M, Matthews A, Scott PA. The impact of the work environment of nurses on patient safety outcomes: a multi-level modelling approach. International journal of nursing studies. 2013;50(2):253–63. 10.1016/j.ijnurstu.2012.08.020 23116681

[65] Linden DVD, Keijsers GP, Eling P, Schaijk RV. Work stress and attentional difficulties: An initial study on burnout and cognitive failures. Work & Stress. 2005;19(1):23–36.

