# Supplementary material for: Healthcare Staff Wellbeing, Burnout, and Patient Safety: A Systematic Review
Source: PLoS One. 2016 Jul 8;11(7):e0159015. doi: 10.1371/journal.pone.0159015 (PMC4938539; doi:10.1371/journal.pone.0159015)
Supplement: S1 Table — (PDF) [file pone.0159015.s001.pdf]

First author	Year	Country	Design	Sample	Wellbeing Measure(s)	Burnout Measure	Patient Safety Measure(s)	Key Findings	Significant correlation?
Arakawa	2011	Japan	Cross-sectional survey	6445 Nurses	SF-36		Self-report medical errors or incidents, accompanied by collaborating information	Role (emotional) but not mental health predicted the occurrence of medical incidents and errors in logistic regression. OR 0.996, (0.993-0.999), p = .007	Partial
Arimura	2010	Japan	Cross-sectional survey	454 Nurses	GHQ (28-item)		Self-report of having been responsible for a medical error in the past month (accident or incident), and had submitted written explanation to the hospital	GHQ score significantly associated with errors (in multivariate analysis). OR 1.1, 1.0-1.1, p < .05. Total score on GHQ, somatic symptoms, anxiety/insomnia, social dysfunction, and depression were all significantly higher in those reporting errors when not controlling for other factors (t-tests).	Yes
Baldwin	1997	Scotland	Prospective, mixed methods	142 Medical students	GHQ (28-item)		Self-reported number of mistakes in the past year, by 3 levels of severity and 3 time periods	No sig. correlation between GHQ (total & subscales) and errors. 'Feeling overwhelmed' correlated with GHQ subscales and errors in past month.	No
Dollarhide	2014	USA	Prospective, longitudinal study	185 Attending physicians (residents and interns)	Emotional stress (From the Diary of Ambulatory Behavioural States, 1998)		Real-time medication event reporting tool (MERT) - self-reporting of medication events (errors and near misses)	Sig. higher emotional stress scores on days in which they reported a medication event (p < .01). Emotional stress scores were approximately 33% higher among 'event reporting' versus 'non-event reporting' physicians (p < .05) across all days of the study.	Yes
Dorrian	2006	Australia	Pilot, survey	23 Nurses	Stress from 'very' to 'not at all', fatigue, mental exhaustion		Frequency, type (out of 6), severity of errors and near errors made OR observed	Stress ratings didn't enter the final model that predicted errors.	No
Dorrian	2008	Australia	Daily-diary	41 Nurses	Stress from 'very' to 'not at all', fatigue, mental exhaustion		Frequency, type (out of 6), severity of errors and near errors made OR observed	Stress ratings (OR = 1.5) (and struggling to stay awake during shift) were significant predictors of error (p < .05).	Yes
Fogarty	2006	Australia	Cross-sectional survey	176 Nurses	Occupational PANAS		Error index; self-reported frequency of medication error in past 12 months	Significant correlation between errors and morale (r = -0.21, p < .01), and distress (r = 0.17, p < .05).	Yes
Hammer	1986	USA	Cross-sectional survey	374 Paramedics	MPSS-R		On-the-job behaviour inventory: 'critical' scale for judgment errors in patient care	Significantly more errors were reported by respondents with less somatic distress (t = -0.14) and less total stress (t = -0.15)	Yes
Houston	1997	England	Prospective cohort study	30 Junior house officers	GHQ (28-item)		Medical errors questionnaire, self-propensity to make errors and their frequency	Only the anxiety/insomnia subscale of the GHQ significantly correlated with errors, and only at Time 2 (r = .40, p < .05).	Partial
Niven	2015	UK	Cross-sectional survey	1205 Pharmacists	Mental strain (depression & anxiety)		Self-report errors (minor and serious) in the previous 4 weeks	Anxiety, but not depression had a significant effect on errors (both minor and serious). Anxiety also mediated the effect of presenteeism on errors.	Partial
Park	2013	Korea	Correlational study	279 Nurses	SF-KOSS (job stress)		First item of the AHRQ patient safety culture survey	Total job stress score significantly correlated with patient safety incidents (r = 0.217). In the multivariate regression, only lack of job autonomy and job instability subscales of job stress had significant effects on incidents	Partial
Pelliciotti	2010	Brazil	Cross-sectional survey	94 Nurses	SF-36		Self-reported medication error in the previous 4 weeks	Those who reported errors had significantly worse mental health (p = .01).	Yes
Saleh	2014	Egypt	Cross-sectional survey	52 Nurses	CES-D		Medication errors observation sheet	Depression was a significant independent predictor of medication errors (β = 0.381). The more depressed, the significantly more errors made (r = 0.62, p < .001).	Yes
Suzuki	2004	Japan	Cross-sectional survey	4279 Nurses	GHQ-12		Experience of errors in previous 12 months	Poor mental health was significantly associated with all types of errors, in all analyses.	Yes
Tanaka	2012	Japan	Prospective cohort study	789 Nurses	NSS, HADS		Self-perceived near misses and AEs in previous 6 months	Depression and Job Stressors' significant association with medical error risk (AEs and near misses) was mediated through decreased attention.	Yes
Wilkins	2008	Canada	Cross-sectional survey	4379 Registered Nurses	Mental Health (1Q)		Self-report medication error in past 12 months	Fair or poor mental health and medication errors had an OR of 1.3 (0.8-2.3) compared to OR of 1 for good, very good, or excellent mental health and errors. This trended on significance but p = 0.075	No
De Oliveira	2013	USA	Cross-sectional survey	1417 Anesthetists	HANDS	MBI-12	Frequency of self-reported errors	Significant association between errors occurring often or multiple times and depression, EE, PA and DP (all p < .001)	Yes
Dugan	1996	USA	Cross-sectional survey	293 Nurses	Stress Continuum Scale (SCS)	Symptom-based stress survey score (has been previously used to measure burnout)	No. of patient falls, medical errors and IV errors occurred during the month of the study, at the unit level. Obtained from hospital records.	Significant correlations between SCS scores and total patient incidents (r = .43), medication errors (r = .40), but not IV errors. Only total patient incidents was significantly correlated with SCS scores at all 3 time points. The higher the hospital unit's mean score on SCS, the significantly higher percentage of patient incidents, (F = 6.08, df = 1, 41, p = .02). No significant associations between symptom-based stress survey and patient incidents of any type.	Partial
Dyrbye	2013	USA	Cross-sectional survey	7288 Physicians (hospital-based and GPs)	Mental QoL PWBI	PWBI (burnout, PA of MBI)	Self-perceived errors in the last 3 months	Physicians who reported a recent error were more likely to endorse each item of the PWBI and a greater number of total items (p < .001 for all).	Yes

First author	Year	Country	Design	Sample	Wellbeing Measure(s)	Burnout Measure	Patient Safety Measure(s)	Key Findings	Significant correlation?
Fahrenkopf	2008	USA	Prospective cohort study	123 Residents	HANDS), self-reported depression, QoL	MBI	Self-reported errors, objective errors (chart review and daily reports)	Depressed pp's made sig. more (objectively measured) errors per month than non-depressed ($p < .001$), but no difference for self-reported errors. Burnt-out residents self-reported more errors than non-burnt-out ($p = .02$), but no difference using objective errors measures ($p = .4$). No mention of QoL and error associations.	Partial
Garrouste-Orgeas	2015	France	Prospective cohort study	1534 hospital staff (physicians, nurses, physio's etc.)	CES-D	MBI (Fontaine French version)	Research assistants collected data (chart audit etc.), plus Safety Attitudes Questionnaire (SAQ-ICU)	MBI and CES-D did not correlate with the SAQ-ICU score. Depression was an independent risk factor for error ($p = .01$), but burnout was not. Burnout was also not significantly associated with adverse events	Partial
Hayashino	2012	Japan	Prospective cohort study	836 Hospital-based practicing physicians	WHO-5	MBI 17 items	Self-perceived errors in the last year	The significant association between burnout (EE and DP) and depression with error was modified by Hope.	Yes
Linzer	2009	USA	Cross-sectional survey	422 Physicians	Job stress scale	Burnout (single item)	Objective error scores (treatment, management and prevention errors) by chart audits	No associations between physician reactions (stress and burnout) and patient care (or total error).	No
Shanafelt	2002	USA	Cross-sectional survey	115 Internal medicine residents	PRIME-MD	MBI (EE & DP)	Self-report suboptimal patient care "I made treatment or medication errors that were not due to a lack of knowledge or inexperience'	Depression was not significantly correlated with patient care practices. Burnt-out residents were significantly more likely to report making treatment or medication errors several times per year, monthly, and weekly than those not burnt-out $p < .05$	Partial
Shanafelt	2010	USA	Cross-sectional survey	7905 Surgeons	PRIME-MD, SF-12	MBI	Self-perceived error in the last 3 months	Reporting an error in the last 3 months was associated with significant increases ($p < .0001$) in EE, the risk for screening positive for depression, and a decline in mental QoL. Similarly, Increases in DP and EE were significantly associated with an increase in the likelihood of reporting an error, and increases in PA and mental QOL were associated with a decrease in the likelihood of reporting an error.	Yes
West	2006	USA	Prospective, longitudinal study	184 Internal medicine trainees	QoL, Depression (2-Q)	MBI	Self-perceived errors in the last 3 months	Error was significantly associated with an increase in EE and positive screening for depression in the subsequent time points. Higher levels of burnout (all domains) were significantly associated with increased odds of reporting an error in the following time points. Reciprocal relationship.	Yes
West	2009	USA	Prospective, longitudinal study	380 Internal medicine trainees	QoL, Depression (2-Q)	MBI	Self-perceived errors in the last 3 months	Diminished QoL, higher levels of burnout (all subscales) and positive screening for depression were each significantly associated with increased odds of reporting errors in the subsequent 3 months.	Yes
Bao	2013	Spain	Cross-sectional survey	234 Nurses		Shirom-Melamed's burnout scale	Accident propensity	Burnout was significantly correlated with accident propensity ($r = .37$, $p < .001$) and it fully mediated the effect of ethical value incongruence on accident propensity.	Yes
Block	2013	USA	Cross-sectional survey	55 First year internal medicine residents		MBI (modified)	Self-reported medical errors, Safety Attitudes Questionnaire	"Higher burnout scores tended to be associated with self-reported errors and poorer reported safety." $p < .001$ for difference between burnout tertiles and SAQ safety scores. $p < .05$ for burnout tertiles and errors made due to workload, and forgetting to convey important information.	Yes
Chen	2013	Taiwan	Cross-sectional survey	839 Physicians (including surgeons)		MBI-GS	"Medical error experience"	The number of medical errors reported was significantly and strongly associated with high-level of EE ($p < .001$)	Yes
Cimiotti	2012	USA	Cross-sectional survey and secondary data analysis	7728 Nurses (estimated number)		EE scale of MBI-HSS	Objective data: Care-associated Surgical Site Infections (SSIs) and Urinary Tract Infections (UTIs)	The staffing-infection relationship was fully mediated by burnout. Burnout was associated with both UTIs ($\beta = .085$, $p = .02$) and SSIs ($\beta = 1.54$, $p < .01$).	Yes
Halbesleben	2008	USA	Cross-sectional survey	148 Nurses		EE and DP of MBI	AHRQ Patient safety culture survey	Higher burnout (EE and DP) was significantly associated with a lower patient safety grade, perceptions of a less safe environment, near-miss reporting frequency, but not with event report frequency.	Partial
Holden	2011	USA	Cross-sectional survey	199 Nurses		EE of MBI	Perceived likelihood of medication error	Burnout was not significantly associated with the likelihood of medication error.	No

First author	Year	Country	Design	Sample	Wellbeing Measure(s)	Burnout Measure	Patient Safety Measure(s)	Key Findings	Significant correlation?
Holden	2010	USA	Cross-sectional survey	79 Pharmacists (and Pharmacy technicians)		EE of MBI	Medication error and adverse event likelihood (single item self-report for each)	Burnout was significantly associated with medication error likelihood (OR = 1.60) and adverse drug event likelihood (OR = 1.52), both $p < .05$.	Yes
Kirwan	2013	Ireland	Cross-sectional survey	1397 Nurses		EE of MBI	AHRQ patient safety grade. Self-report number of formal adverse events they had submitted in the past year	Ward mean for EE didn't significantly contribute to the safety grade ($p = .120$), or to the number of formal adverse event reports ($p = .089$)	No
Klein	2010	Germany	Cross-sectional survey	1311 Surgeon		Copenhagen Burnout Inventory	Self-reported frequency of diagnostic & therapeutic errors	Burnout was only significantly associated with both therapeutic errors (OR = 2.54) and diagnostic errors (OR = 1.94) in male, and not in female surgeons.	Partial
Laschinger	2006	Canada	Cross-sectional survey	8597 Nurses		MBI-HSS	Self-perceived adverse event in past year (falls, nosocomial infections, medical errors, patient complaints)	Burnout partially mediated the relationship between worklife factors and adverse events in the model with best fit. In bivariate analysis, there was a significant correlation between adverse events and EE ($r = .30$), DP ($r = .34$) and PA ($r = -.22$).	Yes
Prins	2009	The Netherlands	Cross-sectional survey	2115 Residents		Utrecht Burn-out Scale	Self-reported errors	Action/inexperience errors were significantly correlated with EE ($r = .20$, $p < .0001$), DP ($r = .29$, $p < .001$), PA ($r = -.05$, $p < .001$). Errors due to lack of time were significantly correlated with EE ($r = .43$), DP ($r = .42$), PA ($r = -.08$), all $p < .001$.Specific error questions were all significantly correlated with EE and DP, and the majority also correlated with PA. Residents with moderate or severe burnout reported sig. more errors than residents without burnout. And those with severe burnout reported sig. more errors due to lack of time than those with moderate burnout.	Yes
Profit	2014	USA	Cross-sectional survey	2073 Hospital staff (physicians, nurse practitioners etc.)		EE of MBI	Safety attitudes questionnaire	NICUs with a greater % of respondents reporting burnout had a smaller % of respondents reporting a good safety climate ($r = -0.38$, $p = .01$). A burnout score of less than 25 (signifying resilience) was significantly associated with safety climate ($r = .60$, $p < .001$).	Yes
Ramanujan	2008	USA	Cross-sectional survey	430 Nurses		Workplace demands': EE and DP of MBI	Self-report 'perception of patient safety' Likert scale questions	In the final model, EE had a -0.056 effect on safety, but only an indirect effect (-0.056) through DP. DP had a -0.189 total effect on safety, which was a direct effect. The model had 'good fit'	Yes
Squires	2010	Canada	Cross-sectional survey	267 Registered Nurses in Acute Care		EE of MBI	Self-report Medication errors, 1Q	In the final model, medication errors lead to EE at a significant level ($p < .05$), but with a small effect size (0.14)<br>EE significantly correlated with medication errors in univariate analysis ($t = 0.22$, $p < .01$) in that higher levels of EE correlated with more medication errors.	Yes
Teng	2010	Taiwan	Cross-sectional survey	458 Nurses		MBI-HSS modified	Frequency of various patient safety indicators (e.g. administrative errors and nosocomial infections)	Significant correlation between patient safety and EE ($r = -.11$, $p < .05$), and DP ($r = -.11$, $p < .05$). Burnout moderated the effects of nursing experience and medical centre on patient safety	Yes
Van Bogaert	2014	Belgium	Cross-sectional survey	1108 Nurses		MBI-HSS	Adverse patient event frequency (including medication errors)	Medication errors were predicted by DP. No association between PA and medication errors. Don't mention EE thus assumed it wasn't related with medication errors.	Partial
Welp	2015	Switzerland	Cross-sectional survey	1425 Nurses and Physicians in ICU		MBI-HSS	Clinician rated patient safety grade (Hospital Survey of Patient Safety Culture)	Correlations: At the individual level, Clinician-rated patient safety significantly correlated with EE ($r = -0.25$, $p < .01$), DP ($r = -0.16$, $p < .01$), and PA ($r = 0.18$, $p < .01$). Multilevel model results: All burnout subscales significantly predicted clinician rated patient safety (BEE = −0.13, $t = -4.52$, $p < 0.001$, BDP=−0.07, $t = -2.11$, $p = 0.04$, BPA = 0.16, $t = 3.38$, $p = 0.002$).	Yes
Williams	2007	USA	Secondary analysis of the MEMO study data	426 Physicians		Burnout (single item)	Likelihood of future error - 9 items	In the mode of best fit, burnout leads to error likelihood, significant at the $p < .05$ level (parameter estimate/intercorrelation = .13).	Yes
Zander	2013	Germany	Longitudinal, cross-sectional survey	4192 Nurses		EE of MBI	PES-NWI; patient safety on the ward	High patient safety was significantly associated with EE in both 1999 ($p = .006$, OR = .572) and in 2009 ($p < .001$, OR = .376). In 2009, high patient safety was a significant factor for lower EE.	Yes

CES-D, Centre for Epidemiological Studies – Depression Scale; GHQ, General Health Questionnaire; MPSS-R, Medical Personnel Stress Survey – Revised; NSS, Nursing Stress Scale; HADS, Hospital Anxiety and Depression Scale; PANAS, Positive And Negative Affect Schedule; SF, Short Form; SF-KOSS, Short Form - Korean Occupational Stress Scale; AHRQ, Agency for Healthcare Research and Quality; HANDS, Harvard National Depression Screening Day Scale; MBI, Maslach Burnout Inventory; EE, Emotional Exhaustion; PA, Personal Accomplishment; DP, Depersonalization; SCS, Stress Continuum Scale; GP, General Practitioner; QoL, Quality of Life; PWBI, Physician Well-Being Index; SAQ-ICU, Safety Attitudes Questionnaire – Intensive Care Unit; WHO, World Health Organisation; PRIME-MD, Primary Care Evaluation of Mental Disorders; SAQ, Safety Attitudes Questionnaire; MBI-GS, Maslach Burnout Inventory – General Survey; MBI-HSS, Maslach Burnout Inventory, Human Services Survey; SSI, surgical site infection; UTI, urinary tract infection; NICU, Neonatal Intensive Care Unit; PES-NWI, Practice Environment Scale – Nursing Work Index
