# Supplementary material for: Healthcare Staff Wellbeing, Burnout, and Patient Safety: A Systematic Review
Source: PLoS One. 2016 Jul 8;11(7):e0159015. doi: 10.1371/journal.pone.0159015 (PMC4938539; doi:10.1371/journal.pone.0159015)
Supplement: S2 File — (PDF) [file pone.0159015.s003.pdf]

Supplementary File 2: Search criteria for Medline (Ovid)

1. *health personnel/ or exp medical staff/ or exp nurses/ or *nursing staff/ or exp physicians/
2. Exp mental health/ or * anxiety disorders/ or exp *depressive disorder, major/
3. Exp Stress, Psychological/
4. Exp Occupational Health/
5. Exp “Quality of Life”/
6. 2 or 3 or 4 or 5
7. Exp medical errors/ or *patient care/
8. Occupation* stress*.mp. [mp=title, abstract, original title, name of substance word, subject heading word, keyword heading word, protocol supplementary concept word, rare disease supplementary concept word, unique identifier]
9. well?being.mp. [mp=title, abstract, original title, name of substance word, subject heading word, keyword heading word, protocol supplementary concept word, rare disease supplementary concept word, unique identifier]
10. exp Patient Safety/
11. exp “Quality of Health Care”/
12. 7 or 8 or 9 or 10 or 11
13. 1 and 6 and 12
14. limit 13 to (english language and (classical article or "corrected and republished article" or journal article or meta analysis or "review" or "scientific integrity review" or systematic reviews)
