# Supplementary material for: Healthcare Staff Wellbeing, Burnout, and Patient Safety: A Systematic Review
Source: PLoS One. 2016 Jul 8;11(7):e0159015. doi: 10.1371/journal.pone.0159015 (PMC4938539; doi:10.1371/journal.pone.0159015)
Supplement: S3 File — (PDF) [file pone.0159015.s004.pdf]

Supplementary File 3: Data Extraction Template

<table>
<tr><td colspan="2">Researcher’s initials:</td><td colspan="2">Date Performed:</td></tr>
<tr><td colspan="4">Publication Details</td></tr>
<tr><td>Author:</td><td>Year:</td><td colspan="2">Country:</td></tr>
<tr><td colspan="4">Title:</td></tr>
<tr><td colspan="4">Stated aim:</td></tr>
<tr><td colspan="4">Study design:</td></tr>
<tr><td colspan="4">Participant Details</td></tr>
<tr><td>Number:</td><td colspan="2">Age:</td><td>Gender:</td></tr>
<tr><td>Roles:</td><td colspan="2">GP Surgery / Hospital</td><td>Partners / Salaried / NA</td></tr>
<tr><td colspan="4">Part time/Full time:</td></tr>
<tr><td colspan="4">Other Info (e.g. Demographics, years worked etc.):</td></tr>
<tr><td>Number of sites:</td><td colspan="3">Geographical locations:</td></tr>
<tr><td colspan="2">Inclusion criteria:</td><td colspan="2">Recruitment method:</td></tr>
<tr><td colspan="4">Measures</td></tr>
<tr><td colspan="4">Wellbeing/Burnout/Both</td></tr>
<tr><td colspan="4">Measures:

Collection method:</td></tr>
<tr><td colspan="4">Patient Safety</td></tr>
<tr><td colspan="4">Measures:

Collection method:</td></tr>
<tr><td colspan="4">Additional measures:

Collection method:</td></tr>
</table>

**Direction of relationship:**
Study Information
**Duration:**
**Follow up duration:**
**Limitations:**
Results
**Statistical analyses:**
**Summary of results *(including correlation coefficient):***
**Effect size *(page number)*:**
**Author's conclusion:**
Accept/Reject
**Reason for exclusion:**
**Reviewer's comments:**
**Overall quality of study:**
