# Supplementary material for: Healthcare Staff Wellbeing, Burnout, and Patient Safety: A Systematic Review
Source: PLoS One. 2016 Jul 8;11(7):e0159015. doi: 10.1371/journal.pone.0159015 (PMC4938539; doi:10.1371/journal.pone.0159015)
Supplement: S4 File — (PDF) [file pone.0159015.s005.pdf]

Supplementary File 4: Quality assessment tool and scoring guide

Criteria	Poor/High risk of bias	Average/Medium risk of bias	Good/Low risk of bias	Unknown/N.A
**Representativeness**	Self-selected sample from one site (and one ward), with a low proportion of eligible participants taking part	Self-selected sample, from more than one ward, with a medium proportion of eligible participants taking part	More than one site, high proportion of eligible participants taking part	
**Randomisation**	Self-selected participants	Recruitment sent to all/random sample of eligible participants, but <50% participated	Recruitment sent to all/random sample of eligible participants, but >50% participated	
**Blinding**	No blinding or incomplete blinding, which is likely to influence the outcome	Attempted blinding, but likely not carried out effectively	Outcome not likely influenced by lack of/broken blinding.<br>Or, effective blinding.	
**Measure of patient safety/quality**	Measure developed for this study, with no mention of validity, reliability or piloting	Measure developed for this study, with attempts to display validation (e.g. concurrent validity)	Validated, well known measure OR new measure with validity and reliability displayed (e.g. more than one type of validity)	
**Measure of wellbeing/burnout**	Measure developed for this study, with no mention of validity, reliability or piloting	Measure developed for this study, with attempts to display validation (e.g. concurrent validity)	Validated, well known measure OR new measure with validity and reliability displayed (e.g. more than one type of validity)	
**Participants lost to follow up/Incomplete outcome data**	Participants lost, but no mention of differences between completers or non-completers.<br><br>No intention to treat analysis on missing data.		Analysis to check for differences between completers and non-completers, with significant differences controlled for in main analysis.<br><br>Intention to treat analysis for missing data	
**Confounding variables**	No evidence of attempting to account for possible confounding variables in analysis (or recruitment)	Accounted for basic potential confounding variables at either recruitment or analysis (e.g. Age, Gender)	Accounted for basic confounding variables and additional potential confounding variables, at either recruitment or analysis (e.g. Years in practice)	
**Power and effect size**	Power analysis reported, with below small effect size	Power analysis reported, with small - medium effect size	Power analysis reported, with medium – large/large effect size	
