# Supplementary figures and images for: Healthcare Staff Wellbeing, Burnout, and Patient Safety: A Systematic Review
Source: PLoS One. 2016 Jul 8;11(7):e0159015. doi: 10.1371/journal.pone.0159015 (PMC4938539; doi:10.1371/journal.pone.0159015)

### Risk of bias/quality assessment graphs

All studies ( $n = 46$ )

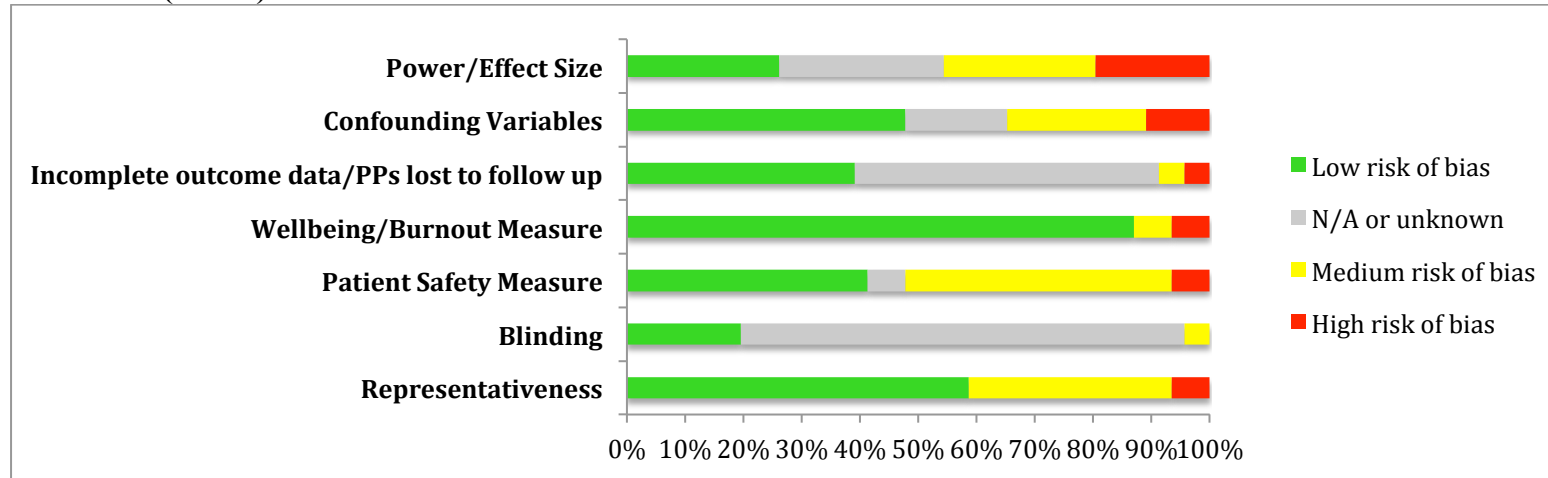

Burnout studies ( $n = 30$ )

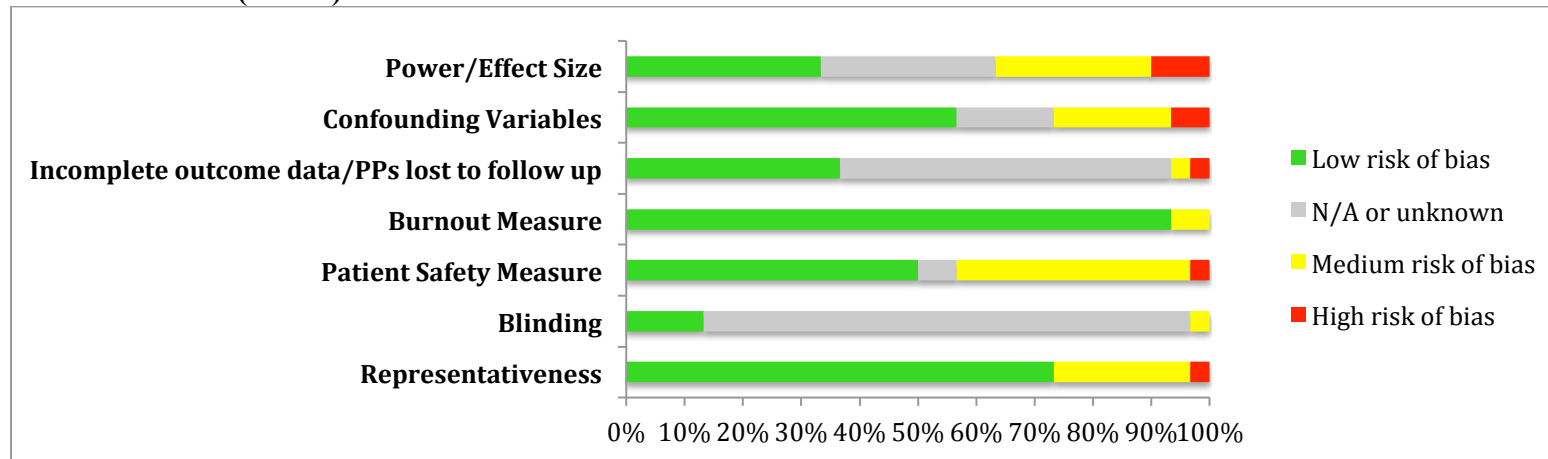

**Wellbeing studies ( $n = 27$ )**

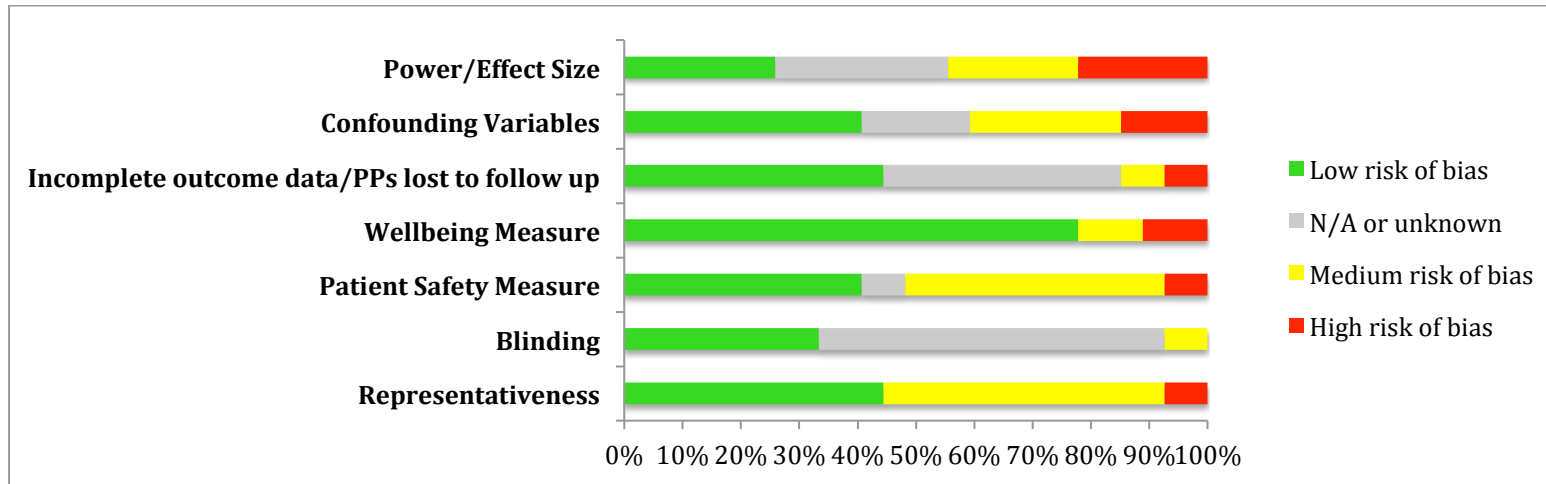

Supplement: S5 File — (PDF) [file pone.0159015.s006.pdf]
